# Cephalosporin-Induced Toxic Epidermal Necrolysis Treated with Intravenous Immunoglobulin

**DOI:** 10.7759/cureus.359

**Published:** 2015-10-21

**Authors:** Konstantin Boroda, Li Li, Louis Riina, Shadab Ahmed

**Affiliations:** 1 Radiology, Stony Brook University; 2 Internal Medicine, Albert Einstein College of Medicine; 3 Internal Medicine, Nassau University Medical Center; 4 Plastic Surgery, Nassau University Medical Center; 5 Infectious Disease, Nassau University Medical Center

**Keywords:** toxic epidermal necrolysis, ten, ivig, stevens johnson syndrome, sjs

## Abstract

Toxic epidermal necrolysis (TEN) is a life-threatening cutaneous reaction to various medications, including antipsychotics and antibiotics. While cephalosporin-induced TEN is very rare, we present a case of cefepime-induced TEN. There are several commonly used therapies for TEN, including immunosuppressive agents and intravenous immunoglobulin (IVIG), but their true efficacy has not been proven. In this case, the patient was treated with IVIG. The role of IVIG as therapy for TEN is currently being investigated. Prior observational studies suggest IVIG infers clinic benefit; however, recent meta-analyses have not shown any benefit. Our patient initially showed clinical improvement with IVIG therapy but, unfortunately, later succumbed to sepsis. We will provide a brief review of the current research of the pathological mechanism of Stevens-Johnson syndrome (SJS)/TEN and the mechanism of action of IVIG specifically in TEN/SJS.

## Introduction

Stevens-Johnson syndrome (SJS) and toxic epidermal necrolysis (TEN) are life-threatening adverse reactions to drugs characterized by epidermal detachment due to keratinocyte death. SJS is defined as the involvement of less than 10% of body surface area while TEN affects more than 30% [1]. Common triggers of TEN/SJS include sulfonamide antibiotics, anticonvulsants, allopurinol, and NSAIDs [2]. We present a case of cefepime-induced TEN.

## Case presentation

**Figure 1:**
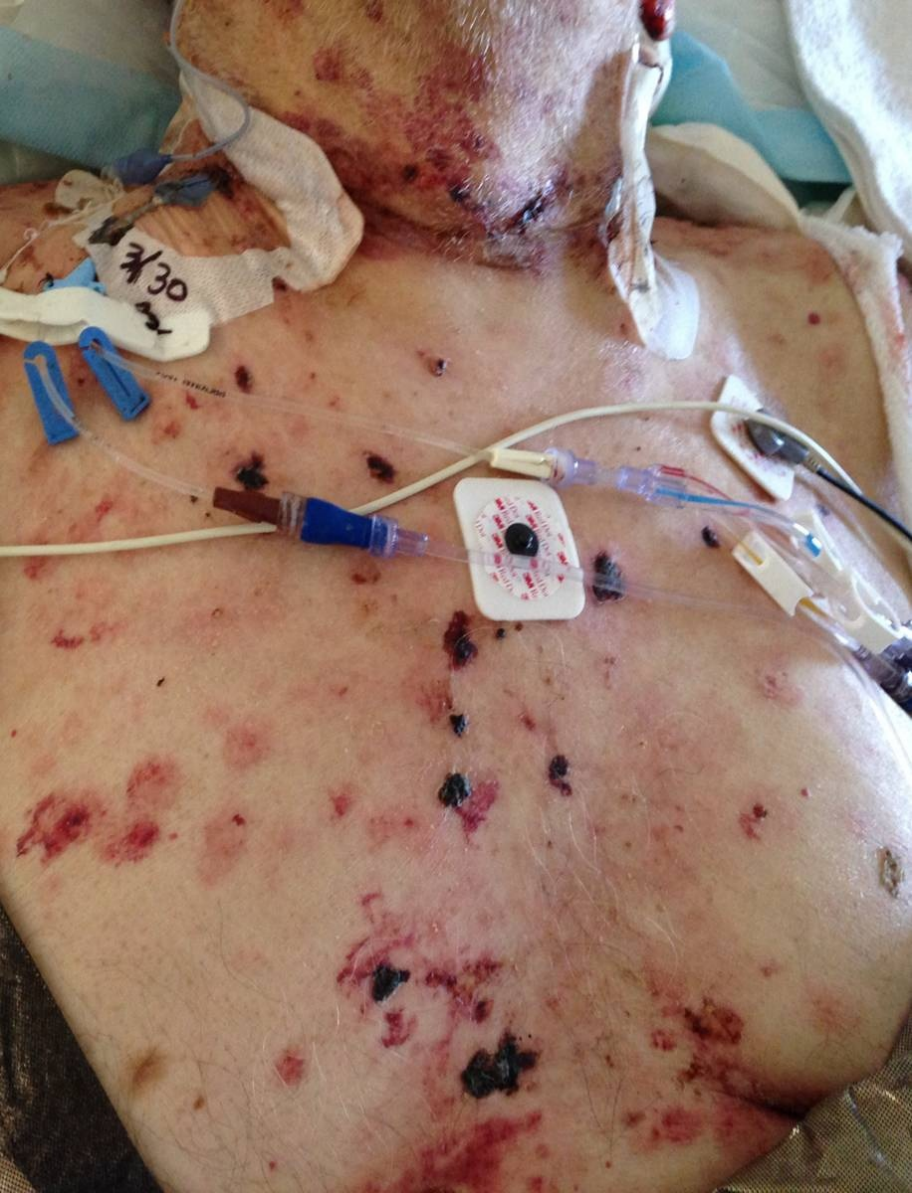
Chest lesions.

An 88-year-old Caucasian male, with a past medical history of hypertension, hepatitis C infection, and prostate cancer, was treated with cefepime and vancomycin for aspiration pneumonia following an open reduction internal fixation procedure for a left hip intertrochanteric fracture. Informed patient consent had been obtained for his treatment. On the second day of antibiotic therapy, the patient developed a bullous rash on his chest and abdomen, as seen in Figure 1.

**Figure 2:**
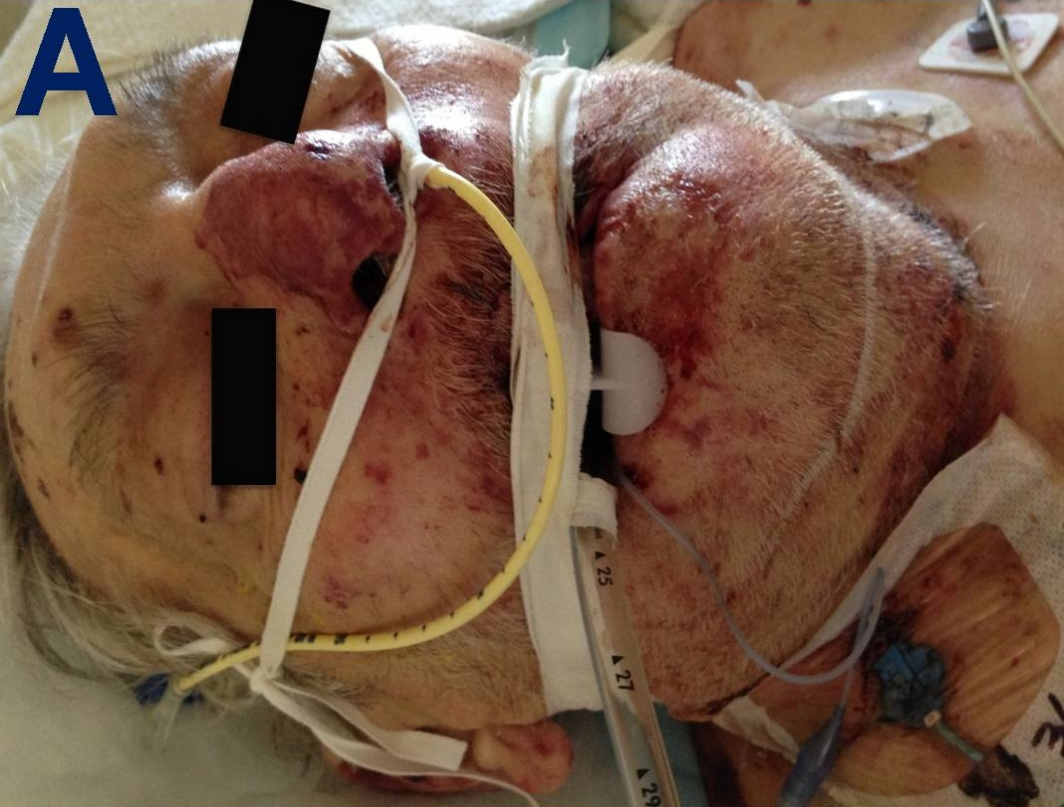
Facial lesions.

SJS-TEN was suspected and cefepime was discontinued immediately. Aztreonam and metronidazole were started instead. The rash continued to spread to his forehead, nose, oral mucosa, and chin (Figure 2).

The rash also spread to his upper and lower extremities; the patient subsequently developed multi-organ failure. Skin biopsy and pathology confirmed the diagnosis of TEN. The patient was given two doses of intravenous immunoglobulin (IVIG) and was subsequently transferred to the hospital’s burn unit while on two vasopressor medications and intubated with a FiO2 of 100%. On physical examination, the patient had the diffuse spread of macular and bullous lesions on his anterior and posterior torso, along with hemorrhagic crusting of bilateral upper and lower extremities, face, and oral mucosa, which correlated to approximately 40% of his total body surface area. While the skin lesions improved over time, he continued to have fevers and leukocytosis with bandemia. Blood, urine, and respiratory bacterial and fungal cultures were negative. He was maintained on empiric antibiotic therapy with tigecycline and metronidazole but succumbed to sepsis on Day 12 of his hospitalization.

## Discussion

TEN carries a 30% mortality rate with infection being the most common cause of death [2]. Severe cutaneous reactions from cephalosporins are very rare, but have been previously documented, usually occurring during coadministration with another antibiotic [3]. Genetic predispositions have been identified, such as associations between carbamazepine-induced SJS/TEN and members of the HLA-B15 family [4]. The underlying mechanism of epidermal damage is a drug-induced immunological reaction mediated by CD8+ cytotoxic lymphocytes and natural killer cells [5]. Cytotoxic proteins, like Fas–FasL, perforin/granzyme B, and granulysin, released from antigen-primed cytotoxic T cells and NK cells cause cell damage and apoptosis [5]. Recent studies have shown granulysin to be a key mediator of keratinocyte apoptosis [5]. It was found in high concentrations in the blister fluid and showed cytotoxicity in in vitro studies and in in vivo injections to the skin of nude mice. Serum granulysin levels are found to rise even before the appearance of skin lesions making it a potential diagnostic target [6]. Another factor that contributes to the extent of epidermal detachment is the impairment of T-reg cells during the acute phase of TEN [7]. While these T-reg cells are found in normal frequencies in blood, they fail to suppress the activation of effector T cells, resulting in epidermal damage [7].

The management of TEN includes wound and ocular care in a burn unit. The role of systemic glucocorticoids is limited to the early stages of the disease. IVIG, plasmapheresis, and cyclosporine have been reported with inconsistent efficacy. A novel approach for management of TEN is to aim at interrupting the apoptotic pathway and the activation of CD8+ cytotoxic lymphocytes and NK cells. Studies have shown that blockage of the TNF-α pathway with the monoclonal antibody, Infliximab, or soluble fusion protein for TNF- α, Etanercept, improves mortality [8-9].

Our patient received IVIG and his skin lesions actually improved, so IVIG may have contributed to this. Unfortunately, the patient expired secondary to sepsis, so the full benefit of IVIG could not be assessed. There have been several proposed immunomodulatory mechanisms for IVIG; they are distinguished by whether they depend on the Fab region or the Fc region of the Ig molecule. Certain mechanisms may function in certain diseases; it is also likely that various mechanisms function simultaneously [10]. TEN is a T cell-mediated disease, and an IVIG mechanism in this disease was proposed by De Groot and colleagues using a mouse model. They have shown that IVIG activates natural regulatory T cells (Tregs), which results in reduced proliferation of effector T cells and suppression of immune response. T cells bind to epitopes on the Fc region of Ig and are then tolerized to antigens that are fused to the Ig; this expands the CD4+CD35+FoxP3+ regulatory T cell pool, which switches the response from immunogenicity to self-tolerance [11-12]. However, this mechanism has not yet been shown in human cells [10].

Although IVIG is commonly used to treat TEN and SJS, its efficacy is uncertain. Several clinical case reports and retrospective studies have found decreased mortality associated with IVIG. However, larger meta-analyses and rigorous statistical analyses showed that IVIG provides no added benefit. The lack of uniformity in previous TEN/SJS study designs assessing disease severity, the dose of IVIG, timing of IVIG administration, and patient comorbidities make the assessment of efficacy very difficult. Additionally, the lack of control groups further decreases sensitivity. There is a need for randomized control trials in order to truly evaluate the efficacy of IVIG [13-16].

The use of IVIG also has side-effects, with hemolysis being more common than previously thought. This side-effect is postulated to be dose-dependent, with generally a mild decrease in hemoglobin and hematocrit, while severe anemia requiring transfusion is not common but can also occur [17-18].

## Conclusions

Overall, the use of IVIG is controversial. In the past, its use in autoimmune disease was highly regarded; however, recently, its efficacy has been brought into question, with several large meta-analyses and retrospective studies showing no added benefit. In this case, IVIG appeared to show improvement of the patient’s skin lesions, although the patient expired due to sepsis. The true value of IVIG can only be reliably assessed via randomized control trials.
